# Urinary Tract Infections in Elderly Patients: A 10-Year Study on Their Epidemiology and Antibiotic Resistance Based on the WHO Access, Watch, Reserve (AWaRe) Classification

**DOI:** 10.3390/antibiotics10091098

**Published:** 2021-09-11

**Authors:** Márió Gajdács, Marianna Ábrók, Andrea Lázár, Katalin Burián

**Affiliations:** 1 Department of Oral Biology and Experimental Dental Research, Faculty of Dentistry, University of Szeged, 6720 Szeged, Hungary; 2 Department of Medical Microbiology, Albert Szent-Györgyi Health Center, Faculty of Medicine, University of Szeged, 6725 Szeged, Hungary; abrok.marianna@med.u-szeged.hu (M.Á.); lazar.andrea@med.u-szeged.hu (A.L.); burian.katalin@med.u-szeged.hu (K.B.)

**Keywords:** urinary tract infections (UTIs), epidemiology, elderly, frail, antimicrobial resistance, MDR

## Abstract

The ageing of the population—especially in developed countries—has brought on many societal challenges and has significantly contributed to the burden on healthcare infrastructures worldwide. Elderly persons (aged ≥ 65 years) are at higher risk for developing UTIs, due to a range of intrinsic and extrinsic risk factors, and they often delay seeking treatment. A retrospective observational study was performed regarding the epidemiology and resistance of UTIs in elderly patients. Identification of the isolates was carried out using VITEK 2 ID/AST and MALDI-TOF mass spectrometry. Antibiotic resistance in these isolates was assessed based on EUCAST guidelines, and were grouped into the WHO AWaRe (Access, Watch, Reserve) classification of antimicrobials. During the 10-year study period, *n* = 4214 (421.4 ± 118.7/year) and *n* = 4952 (495.2 ± 274.6) laboratory-confirmed UTIs were recorded in inpatients and outpatients, respectively. The causative agents showed differentiation among outpatients and inpatients: *Escherichia coli* (48.14% vs. 25.65%; *p* = 0.001), *Enterococcus* spp. (20.15% vs. 21.52%; *p* > 0.05), *Klebsiella* spp. (16.28% vs. 16.26%; *p* > 0.05), *Pseudomonas* spp. (4.40%vs. 13.36%; *p* = 0.001); *Proteus-Providencia-Morganella* group (4.56% vs. 10.96%; *p* = 0.001); *Candida* spp. (0.53% vs. 5.98%; *p* = 0.001); *Citrobacter-Enterobacter-Serratia* group (1.90% vs. 2.71%; *p* < 0.05). Significantly higher resistance rates were observed in inpatient isolates for many Access and Watch antibiotics compared to isolates of outpatient origin; in addition, resistance rates were higher in these uropathogens compared to the previously recorded rates in the region. More care should be taken for the diagnosis and treatment of UTIs affecting elderly patients, as they represent a particularly vulnerable patient population.

## 1. Introduction

Urinary tract infections (UTIs) are one of the commonly encountered infectious pathologies worldwide, with ~120–150 million cases each year, being the most common reason to visit a primary care physician [1]. Community-associated UTIs as well as healthcare-associated UTIs represent a major factor of morbidity worldwide. These infections lead to decreased quality of life in the affected patients and are frequently associated with recurrence or sequelae, even if the appropriate antimicrobial therapy was administered [2,3]. UTIs also lead to a tremendous economic burden (estimated to be around ~5 billion US dollars), which corresponds to the burden on the healthcare infrastructure and the subsequent loss of productivity due to workplace absence [4,5].

Based on the anatomical site affected, UTIs may be classified as upper UTIs (UUTI or pyelonephritis) or lower UTIs (LUTI, which may present as (in decreasing incidence) cystitis, urethritis and prostatitis). In a clinical sense, UTIs may be further divided into uncomplicated and complicated UTIs, depending on the presence of factors facilitating bacterial colonization in the urinary tract (e.g., a structural abnormality) or decreasing therapeutic efficacy [6,7]. Members of the Enterobacterales order are the facultative pathogens most commonly implicated as causative agents for UTIs in the general population. These microorganisms possess an advantageous mix of physiological adaptability and the relevant virulence factors (such as a polysaccharide capsule, urease enzyme, fimbriae, pili) to withstand the sheer forces and to thrive on the urinary epithelium [8]. Overall, *Escherichia coli* (or uropathogenic *E. coli* [UPEC]) is the most frequently isolated species in UTIs [9]; nevertheless, in hospitalized and/or immunosuppressed patients, non-conventional urinary pathogens are increasingly present [10,11]. The antimicrobial therapy of UTIs has become increasingly challenging, due to worrying developments in antimicrobial resistance (AMR) worldwide [12]; clinicians are often left with scarce therapeutic choices, as multidrug-resistant (MDR) bacteria are progressively more common in both community-associated and healthcare-associated UTIs [13]. Extended-spectrum β-lactamase (ESBL)-producing Enterobacterales, carbapenem- and fluoroquinolone-resistant *Pseudomonas* spp. and vancomycin-resistant *Enterococcus* spp. (VRE) are all important representatives of urinary MDR isolates, leading to difficult-to-treat infections [14,15].

With the onset of the latest epidemiological transition, the main disease burden has gradually shifted towards non-communicable diseases, leading to substantial increases in life expectancies [16]. The ageing of the population—especially in developed countries—has brought on many societal challenges and has significantly contributed to the burden on healthcare infrastructures worldwide, as individuals with chronic ailments require life-long therapy and care [17]. Elderly persons (aged 65 or over) are at higher risk for developing UTIs, due to a range of intrinsic and extrinsic risk factors, and they often delay seeking treatment. Intrinsic risk factors include age-related immunological senescence and the presence of underlying conditions leading to immunosuppression (e.g., Type II diabetes, malignancy), urinary incontinence, benign prostate hyperplasia, malnourishment and immobility, while extrinsic risk factors include hospitalization, urinary catheterization and chemotherapy [18,19]. The treatment of UTIs in elderly and frail individuals is further hindered as the use of many antimicrobial groups is discouraged, due to their debilitating adverse events and the pathophysiological features (such as decreased kidney and liver function) of these patients [20].

To facilitate the selection of appropriate antimicrobial therapy, it has been encouraged to develop hospital antibiograms based on available local microbiological data, especially for infections where empirical therapy is prevalent [21]. Nevertheless, the available data on the specific epidemiological characteristics of UTIs in elderly patients is scarce, often evaluated only as a consortium of larger population data [22]. Consequently, the aim of this study was to report on the resistance rates and epidemiology of UTIs affecting patients aged ≥ 65 years in the southern region of Hungary during a 10-year (2008–2017) surveillance period.

## 2. Results

### 2.1. Demographic Characteristics, Sample Types

The affected patients presented with the following characteristics: in the outpatient group, the median age was 75 years (range: 65–96) with a pronounced female dominance (60.3%), while in the inpatient group, the median age was 76 years (65–98), with a pronounced male dominance (71.3%). The detailed age distribution of affected patients is presented in Table 1.

During the 10-year study period, *n* = 4214 (421.4 ± 118.7/year, range: 238–567) and *n* = 4952 (495.2 ± 274.6, range: 220–973) laboratory-confirmed UTIs were recorded in inpatients and outpatients, respectively. The distribution of urine sample types in the two groups were the following: for outpatients, voided (midstream) urine samples were the most relevant (*n* = 3769, 76.11%), followed by catheter-specimen urine (*n* = 1107, 22.35%), first-stream urine (*n* = 28, 0.77%), samples obtained through suprapubic bladder aspiration (*n* = 20, 0.50%) and samples obtained after prostate massage (*n* = 8, 0.27%); for inpatients, catheter-specimen urine samples were the most common (*n* = 2961, 70.27%), followed by midstream urine (*n* = 1190, 28.24%), samples obtained through suprapubic bladder aspiration (*n* = 33, 0.78%) and first-stream urine (*n* = 30, 0.71%), respectively.

### 2.2. Distribution of Relevant Pathogens in the UTIs Affecting Elderly Patients

The causative pathogens of UTIs in elderly patients—corresponding to the 10-year study period—are presented in Table 2 (outpatients) and Table 3 (inpatients). Overall, 53 and 51 distinct bacterial/fungal species were recorded in the outpatient and inpatient group, respectively. In both groups, *E. coli* was the most commonly isolated urinary pathogen; nevertheless, the significance of *E. coli* showed pronounced variance between the two groups (48.14% vs. 25.65%; *p* = 0.001), showing to be less of a primary uropathogen in the inpatient group. *Enterococcus* spp. and *Klebsiella* spp. were isolated in similar rates in both patient groups (20.15% vs. 21.52%; *p* > 0.05, and 16.28% vs. 16.26%; *p* > 0.05, respectively), representing the second and third most common isolate. No relevant differences were seen in the isolation rates of *Acinetobacter* spp. (0.83% vs. 0.33%; *p* > 0.05), members of the *Citrobacter-Enterobacter-Serratia* [CES] group (1.90% vs. 2.71%; *p* > 0.05) and *Staphylococcus* spp. (1.55% vs. 2.25%; *p* > 0.05). On the other hand, some less commonly isolated pathogens were significantly more common in inpatients, such as *Candida* spp. (0.53% vs. 5.98%; *p* = 0.001), members of the *Proteus-Providencia-Morganella* [PPM] group (4.65% vs. 10.96%; *p* = 0.001) and *Pseudomonas* spp. (4.40% vs. 13.36%; *p* = 0.001).

### 2.3. Antimicrobial Resistance Rates of Bacterial Uropathogens Based on the WHO AWaRe Classification

The rates of antibiotic resistance among urinary pathogens affecting elderly patients are presented in Table 4, Table 5, Table 6, Table 7, Table 8 and Table 9; namely, for outpatient isolates, Table 4, Table 6 and Table 8, while for inpatient isolates, Table 5, Table 7 and Table 9, corresponding to the WHO Access, Watch and Reserve classification of antibiotics, respectively. Among the Access antibiotics, there were significant differences seen between the resistance rates of outpatient vs. inpatient samples for AMK (0.7% vs. 4.7%; *p* < 0.05) and NIT (3.6% vs. 30.9%; *p* < 0.001) in *E. coli*, AMK (6.3% vs. 12.8%; *p* < 0.05) and GEN (21.9% vs. 31.8%; *p* < 0.05) in *Klebsiella* spp., and GEN (45.4% vs. 30.3%; *p* < 0.01) in *Pseudomonas* spp (Table 4 and Table 5). While percentages of resistance were generally higher in the inpatient group for the majority of cases, statistically significant differences were not seen for other species-antibiotic pairs.

Rates of methicillin-resistance in *S. aureus* (inferred from the results of the FOX susceptibility test: 15.6% vs. 35.8%; *p* < 0.001), vancomycin-resistance in *Enterococcus* spp. (1.0% vs. 4.6%; *p* < 0.05) and resistance to III. generation cephalosporins (inferred from the results of CEF and CTZ susceptibility results in Enterobacterales (*E. coli*: 13.1% vs. 14.4%, *p* > 0.05; *Klebsiella* spp.: 27.7% vs. 43.1%, *p* < 0.01; CES: 31.9% vs. 34.2%; PPM: 42.0% vs. 68.4%, *p* < 0.01), in addition, resistances against other Watch antibiotics are presented in Table 6 and Table 7. There were significant differences seen between the resistance rates of outpatient vs. inpatient samples for FOS (5.5% vs. 17.4%; *p* < 0.01) and TOB (10.8% vs. 27.4%; *p* < 0.01) in *Klebsiella* spp., TOB (5.3% vs. 14.6%; *p* < 0.05) for PPM and for TOB (40.6% vs. 27.5%; *p* < 0.05), CIP (55.0% vs. 40.1%; *p* < 0.05) and LEV (56.4% vs. 43.7%; *p* < 0.05) for *Pseudomonas* spp. Similar to the Access group, resistance rates for Watch antibiotics were generally higher in the inpatient group, but statistical significance was only shown in the previously mentioned cases.

As seen in Table 8 and Table 9, urinary isolates in the elderly retained susceptibility to Reserve antibiotics (no resistant isolate was detected from either outpatient or inpatient samples). In addition, MDR and XDR rates of urinary isolates are also represented in these Tables: rates of MDR were 19.4% and 40.0% (*p* < 0.001) in *Staphylococcus* spp., 1.2% and 5.4% (*p* < 0.05) in *Enterococcus* spp., 16.6% and 16.7% (*p* > 0.05) in *E. coli*, 35.9% and 53.7% in *Klebsiella* spp., 31.9% and 31.6% (*p* > 0.05) in the *Citrobacter-Enterobacter-Serratia* group, 49.8% and 69.7% (*p* < 0.01) in the *Proteus-Providencia-Morganella* group, and 14.2% and 27.2% (*p* < 0.05) in the outpatient and inpatient groups, respectively. Apart from *Psuedomonas* spp. from inpatient origin (0.4%), no XDR isolates were recorded.

## 3. Discussion

UTIs are the third most common (after respiratory tract infections and gastrointestinal infections) types of infections in human medicine, affecting a significant amount of patients worldwide, irrespective of age, gender and socio-economic status [23]. Nevertheless, elderly individuals are particularly sensitive to the development of UTIs, due to age-related physiological changes, emergence of bacteriuria, high prevalence of comorbidities and frequent hospitalization of these patients [18,19]. The prevalence of UTIs among the community-dwelling elderly patients is estimated at 12–29 per 100 person years at risk, while this number is 44–58 per 100 person years at risk for residents of long-term care facilities [24]. Physicians may treat patients with UTIs, if they are armed with the knowledge of the etiological spectrum and resistance rates of urinary pathogens, specific for the healthcare setting, geographical location and relevant patient population [25]. These data may also be useful in decisions for empirical therapy, if the identification of the pathogen has already been carried out, while susceptibility data are still pending [26]. However, the creation and maintenance of such patient-specific antibiograms for UTIs may be difficult, as there are several patient populations (e.g., males, children, elderly, patients affected by a kidney transplant) for which limited epidemiological data are available [27].

As a part of our study, the characterization of 9166 UTIs (4952 outpatient and 4214 inpatient cases)—affecting individuals 65 years of older—was performed, corresponding to a 10-year study period (2008–2017) in the southern region of Hungary, using the WHO AWaRe selection criteria (a tool intended to be used to support pharmaco-epidemiological studies and drug use monitoring) [28]. Our initial hypotheses were that UTIs affecting elderly patients will present with a more diverse distribution of pathogens, and that relevant uropathogenic bacteria will show higher rates of resistance, compared to the rates observed in the general population. While *E. coli* was the most commonly implicated pathogen in both outpatients and inpatients, the ratio of *E. coli* isolates in inpatients was significantly less numerous compared to outpatients, which may be explained by the fact that many uncommon bacteria were also isolated in notable numbers from inpatient samples. The relevance of enterococci should not be underestimated, as they represented >20% of isolates, and the treatment options for these infections may be especially scarce in cases of extensive resistance. The increasing incidence of enterococci with the advanced age of the patients has been verified by other major studies previously; in addition, there has been evidence showing that patients affected by diabetes and/or metabolic disorder are significantly more susceptible to infections caused by Gram-positive cocci [12,22,29,30]. Additionally, the more precise identification method used in the second part of the study period (2013–2017) may also have had a role in the increased detection of enterococci, as it differentiates these isolates more successfully from other streptococci, which would have been dismissed as contaminants or colonizers by the clinical microbiologist and/or physician.

In addition, members of the PPM group, *Pseudomonas* spp. accounted for >10% while *Candida* spp. accounted for >5% of isolates among inpatients. The management of candiduria and *Candida* urinary tract infections is often a controversial topic, as the relationship between the presence of these yeasts and the patient’s complaints may not be verified [31]; nevertheless, yeasts as true urinary pathogens are far more common in older patients, and hospitalized, severely debilitated individuals [32]. This was first verified in the 1980s, when Platt et al. reported that 26.5% of catheter-associated UTIs (defined as >10^5^ microorganisms) are caused by *Candida* spp [33]. In addition, there have been reports on the increasing prevalence of non-albicans *Candida* species (e.g., *C. glabrata*, *C. guilliermondi*, *C. krusei*, *C. parapsilosis* and *C. tropicalis* among others), which has important therapeutic consequences, as non-susceptibility to the first-line treatment fluconazole is high [34]. The emergence of *Pseudomonas* spp. and members of the PPM group in UTIs should also raise concerns, as these bacteria have a plethora of intrinsic resistance mechanisms, which further limit the available treatment options [35,36].

Significant differences were observed in the resistance rates among inpatient and outpatient isolates for several Access and Watch antibiotics; in addition, our study has highlighted the worrisome developments in the levels of MDR in these isolates, irrespective of origin. Resistance rates of outpatient isolates were shown to be very similar to an earlier report in the same region, which has assessed the susceptibility rates of isolates originating from patients visiting the Emergency Department [37]. Overall, the non-susceptibility rates of elderly isolates were comparable to or somewhat higher than (2–10%) previously reported rates in UTIs in the same geographical region [9]; higher rates of resistance were mostly seen for nitrofurantoin, fosfomycin, trimethoprim-sulfamethoxazole, all relevant antibiotics in the therapy of uncomplicated UTIs [38]. Similarly higher rates of resistance were seen for the fluoroquinolones across all analyzed species, a drug group, which has—until recently—been extensively used by primary care physicians in Hungary to treat UTIs [39]. Interestingly, resistance rates to III. generation cephalosporins, trimethoprim-sulfamethoxazole and the fluoroquinolones in *E. coli* were lower (by 5–15%) in isolates originating from the elderly, compared to previously published reports [9].

Since the early 2000s, the prevalence of Enterobacterales resistant to III. generation cephalosporins (most frequently due to the production of ESBLs) in UTIs has shown an increasing an trend worldwide, which has led to the substantial use of carbapenem antibiotics to manage these infections [40,41]. However, carbapenem-resistance (CR) in urinary pathogens is emerging threat, which severely limits the therapeutic arsenal of clinicians to provide safe antimicrobial therapy, often forcing them to use older drugs with a disadvantageous side effect profile (e.g., colistin) or newer, significantly more expensive antibiotics (e.g., meropenem/vaborbactam, if appropriate) with limited availability and clinical experience [42,43,44]. Among the members of Enterobacterales, CR is most common in *K. pneumoniae* and *E. coli*, while among non-fermenters, non-susceptibility of *A. baumannii* complex and *P. aeruginosa* have also shown worrisome developments [45]. A retrospective (2014–2019) study by Shields et al. found that the prevalence of CR pathogens in UTIs was 4.4% from assessing the data of >700 US hospitals [46]. Additionally, their study noted that CR-UTIs were more common in patients aged over 65, and were associated with adverse clinical outcomes (higher rates of subsequent bacteremia, longer hospital stay and recurrence). Due to the reportedly high prevalence of *Enterococcus* spp., one should prepare for the possibility of encountering vancomycin-resistant variants (VRE), with a global prevalence ranging between 0–18% (5–30% in Intensive Care Units) [47]: while in case of *E. faecalis*, susceptibility to ampicillin (and other β-lactams) and aminoglycosides is usually retained, *E. faecium* is highly resistant to these drugs, therefore guidelines recommend therapy with either linezolid or daptomycin, depending on the clinical situation [48].

The number of available reports on the epidemiology of UTIs in the elderly is scarce. Hrbacek et al. reported on the resistance of uncommon urinary isolates, mainly members of the CES (*Citrobacter-Enterobacter-Serratia*) and PPM (*Proteus-Providencia-Morganella*) groups, *Acinetobacter* and *Pseudomonas* [10]: the overall prevalence of these pathogens was 4.65% in a 9-year (2011–2019) study period, with male patients being disproportionally more affected (76.8%); the mean age of patients were 70.3 and 69.2 years for males and females. The majority (50–80%) of the surveyed isolates were resistant to aminopenicillins and I-II. generation cephalosporins, while resistance rates to III. generation cephalosporins, fluoroquinolones, aminoglycosides, carbapenems, nitrofurantoin and SXT were around 5–15%, 20%, 6–33.3%, 10%, 90% and 10–30%, respectively [10]. The increased diversity of urinary pathogens in patients with advanced age was further verified by the findings of Kot et al., noting the increasing importance of *Enterococcus* spp. and *P. mirabilis* as uropathogens in Polish patients [7]. In a rare study focusing on the 10-year epidemiology of suprapubic bladder aspirates, Gajdács et al. found that around half of the samples originated from patients aged ≥ 65; out of these samples, almost one-third (32.6%) were culture-positive (defined as ≥10^2^ CFU/mL), with *Enterococcus* spp., *E. coli* and *Klebsiella* spp. being the three most common isolates [49]. Interestingly, this study also found strict anaerobic bacteria (namely *Finegoldia magna*, *Peptococcus niger*, and *Peptinophilus indolicus*) as relevant pathogens.

Ioannou et al. performed a 3-year retrospective study, during which *n* = 204 UTIs were recorded in a Greek patient population (mean age: >83 years, 61.3% female). The principal pathogen was *E. coli* (40.5%, among which 16.9% were ESBL-producers), while *P. mirabilis*, *P. aeruginosa* and enterococci were all among the more commonly isolated species [18]. In their cohort, 25% of patients had Type II diabetes, 43.6% presented with sepsis, and the overall mortality rate was 17.8%. In an observational study by Artero et al., the relevance of simultaneous bacteremia during a UTI was observed in elderly Spanish patients (*n* = 333, mean age: 81.6); in this study, *E. coli* was the major urinary pathogen (66.9%). Setting in-hospital mortality as their primary outcome measure, they concluded that the presence of bacteremia (in 41.1% of cases) was not associated with longer hospital stays or higher mortality rates (8.8% vs. 9.7%) [50]. Kofteridis et al. assessed the differences in the outcomes of pyelonephritis in older adults with or without diabetes: their cohort consisted of 88 patients with diabetes and 118 controls, with a median age of 74 years in both groups. The most common etiological agents was *E. coli*, but *Candida* spp. was five times more common in patients with diabetes [51]. Compared to the control group, patients with diabetes had worse outcomes in every outcome measure studied (fever, hospitalization, mortality). In a long-term epidemiological study (2001–2018) López-de-Andrés et al. highlighted the importance of age and comorbidities (Type 2 diabetes) in the development of UTIs: the incidence of these infections was 2.14-times higher in non-diabetic males aged 75–84 years, compared to patients aged 18–50 years; while in patients affected by this metabolic disease, UTI incidence was 17.54-times higher in males and 15.47-times higher in females aged 75–84 years, compared to patients aged 18–50 years [52]. In a recent study by Serretiello et al., a five-year (2015–2019) study was conducted on the epidemiology of UTIs in an Italian university hospital: out of the 46,382 patients enrolled in their study, 21.3% (*n* = 9896) were positive for microbial growth, with a 62.2% female patient population. Among the uropathogens, *E. coli* (48.9%) and *K. pneumoniae* (14.9%) were the most numerous, while 13.8% were Gram-positive cocci, with *E. faecalis* being a representative of the group (9.7%). The authors concluded that *Klebsiella* spp. and *E. faecalis* showed the highest levels of resistance in the region, while carbapenems (for Gram-negative bacteria), vancomycin (for Gram-positive bacteria) and WHO Reserve antibiotics are still safe and effective therapeutic alternatives [53].

On the basis of our results, and the results of other study groups described previously, more care should be taken for the diagnosis and treatment of UTIs affecting elderly patients, as they represent a particularly vulnerable patient population [18,24]. The use of various antiseptic solutions (e.g., povidone-iodine, chlorhexidine gluconate) before indwelling urinary catheterization may provide significant benefits in reducing the number of healthcare-associated UTIs [54,55]. Further investigations—particularly to develop specific antibiograms with the aim of aiding local antimicrobial stewardship initiatives—are recommended, and clinicians should be aware of the possible hallmarks of treatment failure in these patients [56].

## 4. Materials and Methods

### 4.1. Study Site, Study Design, Data Collection

The present retrospective observational study was to assess the burden of UTIs in elderly patients in the southern region of Hungary, including the epidemiology and resistance rates of these infections. Based on the data of the Hungarian Central Statistical Office (KSH), the population of Hungary is estimated to be 9,730,772 (population density ~105.1/km^2^), out of which, 20.31% (estimated to be around 1,976,666) are citizens aged 65 or older; males are underrepresented among the country’s elderly (38.30%) [57]. Our study was performed at the Department of Microbiology (previously: Institute of Clinical Microbiology), Albert Szent-Györgyi Health Center (University of Szeged), which is a ~1800-bed primary- and tertiary-care teaching hospital, serving as the major health care facility in the region for ~400,000 patients [58]. The present study was carried out using data collected representing a time period of 10 years (1 January 2008–31 December 2017) at the Department of Microbiology, which is the principal clinical microbiology laboratory of the Health Center. Data collection was performed via an electronic record search in the laboratory information system, corresponding to urine samples (originating from patients aged ≥ 65 years) positive for relevant pathogens, according to the criteria below.

Samples with clinically significant colony counts for suspected urinary pathogens (10^5^ < colony forming units [CFU]/mL; nevertheless, this was subject to interpretation, based on the information provided on the request forms for microbiological analysis and relevant clinical guidelines) were included in the data analysis [9,14]. Only the first isolate per patient was included in the study, while isolates with different antibiotic-susceptibility patterns were considered as different individual isolates [9,14]. In addition, patient data were also collected, that were limited to demographic characteristics (age and sex) and inpatient/outpatient status. Affected patients were grouped into distinct age groups, based on the criteria of the WHO World (WHO 2000–2025) standard population [59].

The study was designed and performed in accordance with the Declaration of Helsinki.

### 4.2. Identification of Bacterial Isolates

The workup of urine samples arriving at the Department of Medical Microbiology (previously: Institute of Clinical Microbiology) was carried out based on the guidelines published by the Hungarian Ministry of Health [60]. Namely, 10 µL of each uncentrifuged urine sample was cultured on a UriSelect chromogenic agar plate (Bio-Rad, Berkeley, CA, USA) with a calibrated loop, according to the manufacturer’s instructions. Plates were incubated for 24–48 h aerobically at 37 °C. If the growth of relevant pathogens was observed in significant colony counts, plates were passed on for identification and antimicrobial susceptibility testing [9]; in case of yeasts or uncommon pathogens, additional procedures were used. In the first five years (2008–2012) of the study, biochemical methods and the VITEK 2 ID/AST automated system (bioMérieux, Marcy-l’Étoile, France) were used for identification (ID); following 2012, matrix-assisted laser desorption/ionization time-of-flight mass spectrometry (MALDI-TOF MS) was also introduced into the routine workflow. During ID, a MicroFlex MALDI Biotyper (Bruker Daltonics, Bremen, Germany) was used, while for spectrum analysis, the MALDI Biotyper RTC 3.1 software and the MALDI Biotyper Library 3.1 (Bruker Daltonics, Bremen, Germany) were utilized. During MALDI-TOF measurements, bacterial cells from overnight cultures were transferred onto a stainless steel target via a sterile toothpick. An on-target extraction was performed by adding 1 µL of 70% formic acid prior to the matrix. After drying at an ambient temperature, the cells were covered with 1 µL matrix (α-cyano-4-hydroxy cinnamic acid in 50% acetonitrile/2.5% trifluoro-acetic acid; Bruker Daltonics, Bremen, Germany). Mass spectrometry measurements were carried out in positive linear mode across the *m*/*z* range of 2 to 20 kDa; for each spectrum, 240 laser shots at 60 Hz in groups of 40 shots per sampling area were collected [61]. Based on spectra analysis, the system provided a log(score) value, indicating the reliability of the MALDI-TOF MS identification: scores < 1.69 showed unreliable identification, 1.70–1.99 corresponded to probable genus-level identification, 2.00–2.29 corresponded to reliable genus-level identification, while a score ≥ 2.30 corresponded to reliable species-level identification [61].

### 4.3. Antimicrobial Susceptibility Testing of Relevant Isolates

Antimicrobial susceptibility testing for relevant bacterial pathogens and the interpretation of the results was performed based on the recommendations of the European Committee on Antimicrobial Susceptibility Testing (EUCAST) at the time of isolation [62]. Susceptibility data were collected for the following antibiotics (when relevant): amikacin (AMK), ampicillin (AMP), azithromycin (AZI), cefepime (FEP), cefoxitin (FOX), ceftriaxone (CEF), ceftaroline-fosamil (CFT), ciprofloxacin (CIP), clindamycin (CLI), colistin (COL), ceftazidime (CTZ), fosfomycin (FOS), fusidic acid (FAA), gentamicin (GEN), imipenem (IMP), levofloxacin (LEV), linezolid (LIN), meropenem (MER), nitrofurantoin (NIT), trimethoprim-sulfamethoxazole (SXT), tigecycline (TIG), quinpristin-dalfopristin (QPD) and vancomycin (VAN), taking into account the intrinsic resistance mechanisms of isolated bacteria [63]. Advanced resistance mechanisms (e.g., resistance to III. generation cephalosporins, methicillin-resistance) were inferred from results of phenotypic tests [14]. The following strains were used as quality controls: *Staphylococcus aureus* ATCC 29213. *Enterococcus faecalis* ATCC 29212. *Escherichia coli* ATCC 25922 and *Pseudomonas aeruginosa* ATCC 27853.

Intermediate results were grouped with and reported as resistant [9,14]. Classification of bacterial isolates as multidrug resistant (MDR) or extensively drug resistant (XDR) was based on the Magiorakos et al. [64]. Antibiotics were classified as “Access”, “Watch” or “Reserve”, based on the World Health Organization (WHO) AWaRe classification 2019 [28].

### 4.4. Statistical Analysis

Descriptive statistical analysis (including means or medians with ranges and percentages to characterize data) was performed using Microsoft Excel 2013 (Microsoft Corp. Redmond, WA, USA). Statistical analyses were performed with SPSS software version 22 (IBM Corp., Endicott, NY, USA), using the χ^2^-test, Student’s t-test and Mann–Whitney U test. The normality of variables was tested using the Kolmogorov–Smirnov test. *p* values < 0.05 were considered statistically significant.

### 4.5. Limitation of the Study

The present study possesses some limitations, which must be acknowledged: a retrospective study design, risk of selection bias (as the study was performed in a tertiary-care hospital, with a high throughput of severely ill patients); limited clinical data to assess the independent risk factors affecting the emergence of UTIs in patients aged ≥ 65 years; due to the change in the identification methods in the second part of the study (2013–2017), the pathogenic spectrum has seemingly broadened, which cannot be verified as these methods were initially (2008–2017) not available; not all antibiotics were routinely tested during susceptibility testing, therefore those results could not be reported to more precisely ascertain MDR/XDR status of the bacteria; and molecular testing was not performed to ascertain the exact mechanism of action behind the phenotypic resistance of individual isolates.

## Figures and Tables

**Table 1:** Age distribution of the affected patients in the outpatient and inpatient groups (2008–2017).

Age Range	Outpatients (*n*, %)	Inpatients (*n*, %)
65–69 yrs	1119 (22.59%)	840 (19.93%)
70–74 yrs	1101 (22.23%)	919 (21.80%)
75–79 yrs	1091 (22.03%)	1008 (23.92%)
80–84 yrs	872 (17.61%)	815 (19.34%)
85 yrs or older	769 (15.54%)	632 (15.01%)

**Table 2:** Distribution of relevant pathogens from outpatient UTIs affecting elderly patients (2008–2017).

	2008	2009	2010	2011	2012	2013	2014	2015	2016	2017	Overall (*n*, %)	
***Acinetobacter* spp.**											**41**	**0.83**
*A. baumannii*					3		4		2	3	12	0.24
*A. haemolyticus*								1			1	0.02
*A. johnsonii*						1	1	2	3	1	8	0.16
*A. junii*						1		2	2	2	7	0.14
*A. lwoffii*	2		1	1	1			1			6	0.12
*A. nosocomialis*								1			1	0.02
*A. pittii*						1		1		1	3	0.06
*A. schindleri*						1					1	0.02
*A. tjernbergiae*								1			1	0.02
*A. ursingii*								1			1	0.02
*Burkholderia cepacia*	1		1			3					5	0.10
***Candida* spp.**											**26**	**0.53**
*C. albicans*			1		1		3	7	2	1	15	0.30
*C. glabrata*							1				1	0.02
*C. krusei*										1	1	0.02
*C. parapsilosis*								1	3		4	0.08
*C. tropicalis*			1	1		2	1				5	0.10
***Citrobacter-Enterobacter-Serratia* group**											**94**	**1.90**
*C. braakii*				1							1	0.02
*C. freundii*	1		1			2				2	6	0.12
*C. koseri*	1	1		1	1	3	3	3	3	6	22	0.44
*Comamonas testosteroni*			1								1	0.02
*E. asburiae*						1	1		1		3	0.06
*E. cloacae*	3	6	3	2	4	4		8	8	4	42	0.85
*E. hormaechei*							4				4	0.08
*E. kobei*						1	1	3		1	4	0.08
***Enterococcus* spp.**											**998**	**20.15**
*E. avium*						1		1	1		3	0.06
*E. faecalis*	58	64	66	41	46	73	109	176	139	188	960	19.39
*E. faecium*	2	1		3	3	3	1	6	7	8	34	0.69
*E. gallinarum*										1	1	0.02
***Escherichia coli***	**153**	**121**	**201**	**94**	**83**	**275**	**337**	**547**	**438**	**135**	**2384**	**48.14**
***Klebsiella* spp.**											**806**	**16.28**
*K. aerogenes*	1	1	3	2	1	2	1	3	2	5	21	0.42
*K. oxytoca*	5	4	9		4	8	13	13	6	14	76	1.53
*K. pneumoniae*	16	48	68	34	29	77	80	106	183	65	706	14.26
*K. variicola*										3	3	0.06
***Proteus-Providencia-Morganella* group**											**226**	**4.56**
*M. morganii*			1	2	2	1		3	3	3	15	0.30
*P. hauseri*										3	3	0.06
*P. mirabilis*	5	2	3	10	14	17	22	33	35	46	187	3.78
*P. vulgaris*			3		2		4	2	4		15	0.30
*Ralstonia picketti*					1	1					2	0.04
*P. rettgerii*								1	1		2	0.04
*P. stuartii*										4	4	0.08
***Pseudomonas* spp.**											**218**	**4.40**
*P. aeruginosa*	21	19	19	25	20	24	18	20	23	21	210	4.24
*P. putida*							1	1	4		6	0.12
*P. stutzeri*			1				1				2	0.04
*Raoultella ornythiolitica*						1		1			2	0.04
*S. marcescens*		1						1	5	1	8	0.16
***Staphylococcus* spp.**											***77***	***1.55***
*S. aureus*	3	2	4	2	4		5	9	24	12	65	1.31
*S. hominis*	1				2		1				4	0.08
*S. lugdunensis*									1		1	0.02
*S. saprophyticus*			2		2			2	1		7	0.14
*Stenotrophomonas maltophilia*				1				1		1	3	0.06
*Streptococcus agalactiae*	4	6	8		2		8	14	16	10	68	1.37
*Ureaplasma urealyticum*	1					1	1	1	1		5	0.10
**Overall**	**278**	**276**	**397**	**220**	**225**	**504**	**621**	**973**	**918**	**542**	**4952**	

**Table 3:** Distribution of relevant pathogens from inpatient UTIs affecting elderly patients (2008–2017).

	2008	2009	2010	2011	2012	2013	2014	2015	2016	2017	Overall (*n*, %)	
*Achromobacter denitrificans*									1		1	0.02
***Acinetobacter* spp.**											**17**	**0.33**
*A. baumannii*	1		2		1	1		2	1	2	10	0.24
*A. junii*								1	1		2	0.05
*A. pittii*								1	1		2	0.05
*Aeromonas salmonicida*			1								1	0.02
*Burkholderia cepacia*	8	1	2	4	2		2		2	1	22	0.52
***Candida* spp.**											**252**	**5.98**
*C. albicans*	4	9	12	3	7	14	26	24	21	42	162	3.84
*C. glabrata*		3	1	2	2		4	17	5	8	42	1.00
*C. guilliermondii*							2		2		4	0.09
*C. inconspicua*				1							1	0.02
*C. kefyr*					1	1	1				3	0.07
*C. krusei*					3		1	1	1		6	0.14
*C. lusitaniae*						4				1	5	0.12
*C. parapsilosis*	1				1			5		3	9	0.21
*C. tropicalis*	2	2	1		3	2	6	4			20	0.47
***Citrobacter-Enterobacter-Serratia* group**											**114**	**2.71**
*C. farmeri*								1			1	0.02
*C. freundii*	2		1	1							4	0.09
*C. koseri*		1	1			3	2	3	3	3	16	0.38
*Corynebacterium urealyticum*									1		1	0.02
*E. asburiae*								1			1	0.02
*E. cloacae*	6	6	16	4	5	11	7	3	6	4	68	1.61
*E. kobei*								3		3	6	0.14
*E. ludwigii*						1		1		2	4	0.09
***Enterococcus* spp.**											**907**	**21.52**
*E. avium*									1		1	0.02
*E. faecalis*	59	46	58	78	73	100	100	93	109	104	820	19.46
*E. faecium*	3	11	5	5	5	11	8	7	19	12	86	2.04
*E. gallinarum*										1	1	0.02
***Escherichia coli***	**61**	**67**	**103**	**110**	**105**	**111**	**147**	**138**	**139**	**100**	**1081**	**25.65**
***Klebsiella* spp.**											**685**	**16.26**
*K. aerogenes*	2	2	3	4	1	1	4	1	1	1	20	0.47
*K. oxytoca*	3	2	1	7	4	6	11	8	9	9	60	1.42
*K. pneumoniae*	24	32	56	49	57	93	80	67	84	63	605	14.36
***Proteus-Providencia-Morganella* group**											**462**	**10.96**
*Kocuria kristinae*						2	1				3	0.07
*M. morganii*	1	1		1		3	2	5	7	5	25	0.59
*Pantoea agglomerans*							2				2	0.05
*P. hauseri*										1	1	0.02
*P. mirabilis*	14	13	25	38	28	48	59	67	51	41	384	9.11
*P. vulgaris*	1	5	6	8	2	5	8	3	6	2	46	1.09
*P. rettgerii*									2	2	4	0.09
*P. stuartii*									1	1	2	0.05
***Pseudomonas* spp.**											**563**	**13.36**
*P. aeruginosa*	34	50	48	52	59	59	73	52	62	68	557	13.22
*P. mosselii*										1	1	0.02
*P. putida*							2	1		2	5	0.12
*S. marcescens*	1				2		2		1	7	13	0.31
***Staphylococcus* spp.**											**95**	**2.25**
*S. aureus*	5	10	3	11	7	13	7	7	8		71	1.68
*S. haemolyticus*	1					1			1		3	0.07
*S. hominis*	1		1	2	3	2					9	0.21
*S. intermedius*	1										1	0.02
*S. saprophyticus*	1						2				3	0.07
*S. maltophilia*		1		1	1	1		2		2	8	0.19
*S. agalactiae*		3		3	2	1				2	11	0.26
**Overall**	**238**	**266**	**350**	**385**	**392**	**507**	**567**	**526**	**554**	**492**	**4214**	

**Table 4:** Percentage of resistant isolates from outpatient UTIs against WHO Access antibiotics.

	**AMK**	**AMP**	**CLI**	**GEN**	**NIT**	**SXT**
*Staphylococcus* spp. (*n* = 77)	10.3% (*n* = 8)	92.2% (*n* = 71)	33.8% (*n* = 26)	3.4% (*n* = 3)	0% (*n* = 0)	16.9% (*n* = 13)
*Enterococcus* spp. (*n* = 998)	n.r.	1.2% (*n* = 12)	n.r.	29.7% (*n* = 296)	1.3% (*n* = 13)	n.r.
*Escherichia coli* (*n* = 2384)	0.7% (*n* = 12)	58.5% (*n* = 1396)	n.r.	8.2% (*n* = 196)	5.6% (*n* = 134)	32.9% (*n* = 784)
*Klebsiella* spp. (*n* = 806)	6.3% (*n* = 51)	n.r.	n.r.	21.9% (*n* = 177)	n.r.	30.1% (*n* = 243)
*Citrobacter-Enterobacter-Serratia* group (*n* = 94)	5.3% (*n* = 5)	n.r.	n.r.	14.9% (*n* = 14)	n.r.	27.7% (*n* = 26)
*Proteus-Providencia-Morganella* group (*n* = 226)	3.1% (*n* = 7)	n.r.	n.r.	21.7% (*n* = 49)	n.r.	64.1% (*n* = 125)
*Pseudomonas* spp. (*n* = 218)	21.6% (*n* = 47)	n.r.	n.r.	45.4% (*n* = 99)	n.r.	n.r.

Abbreviations: amikacin (AMK), ampicillin (AMP), clindamycin (CLI), gentamicin (GEN), nitrofurantoin (NIT), trimethoprim-sulfamethoxazole (SXT), n.r.: not relevant.

**Table 5:** Percentage of resistant isolates from inpatient UTIs against WHO Access antibiotics.

	AMK	AMP	CLI	GEN	NIT	SXT
*Staphylococcus* spp. (*n* = 95)	16.8% (*n* = 16)	97.9% (*n* = 93)	68.4% (*n* = 65)	10.5% (*n* = 10)	6.3% (*n* = 6)	20.0% (*n* = 19)
*Enterococcus* spp. (*n* = 907)	*n*.r.	2.6% (*n* = 24)	n.r.	39.3% (*n* = 356)	5.8% (*n* = 53)	n.r.
*Escherichia coli* (*n* = 1081)	4.7% (*n* = 51)	54.6% (*n* = 590)	n.r.	9.3% (*n* = 101)	30.9% (*n* = 335)	25.2% (*n* = 273)
*Klebsiella* spp. (*n* = 685)	12.8% (*n* = 88)	n.r.	n.r.	31.8% (*n* = 218)	n.r.	27.8% (*n* = 274)
*Citrobacter-Enterobacter-Serratia* group (*n* = 114)	6.1% (*n* = 7)	n.r.	n.r.	17.5% (*n* = 20)	n.r.	28.1% (*n* = 32)
*Proteus-Providencia-Morganella* group (*n* = 462)	5.6% (*n* = 26)	n.r.	n.r.	14.1% (*n* = 65)	n.r.	64.5% (*n* = 298)
*Pseudomonas* spp. (*n* = 563)	16.2% (*n* = 91)	n.r.	n.r.	30.3% (*n* = 171)	n.r.	n.r.

Abbreviations: amikacin (AMK), ampicillin (AMP), clindamycin (CLI), gentamicin (GEN), nitrofurantoin (NIT), trimethoprim-sulfamethoxazole (SXT), n.r.: not relevant (antibiotics affected by intrinsic resistance mechanisms).

**Table 6:** Percentage of resistant isolates from outpatient UTIs against WHO Watch antibiotics.

	AZI	FOX	CEF	CTZ	FEP	CIP	LEV	FOS	FAA	IMP	MER	RIF	TOB	VAN
*Staphylococcus* spp. (*n* = 77)	35.0% (*n* = 27)	15.6% (*n* = 12)	n.r.	n.r.	n.r.	44.1% (*n* = 34)	44.1% (*n* = 34)	n.t.	0% (*n* = 0)	15.6% (*n* = 12)	15.6% (*n* = 12)	0% (*n* = 0)	10.3% (*n* = 8)	0% (*n* = 0)
*Enterococcus* spp. (*n* = 998)	n.r.	n.r.	n.r.	n.r.	n.r.	34.1% (*n* = 340)	34.1% (*n* = 340)	n.r.	n.r.	1.2% (*n* = 12)	n.r.	n.r.	n.r.	1.0% (*n* = 10)
*Escherichia coli* (*n* = 2384)	n.r.	n.r.	13.1% (*n* = 312)	13.1% (*n* = 312)	11.9% (*n* = 285)	40.9% (*n* = 975)	40.9% (*n* = 975)	0.2% (*n* = 4)	n.r.	0% (*n* = 0)	0% (*n* = 0)	n.r.	3.5% (*n* = 83)	n.r.
*Klebsiella* spp. (*n* = 806)	n.r.	n.r.	27.7% (*n* = 223)	27.7% (*n* = 223)	24.7% (*n* = 199)	48.1% (*n* = 388)	48.1% (*n* = 388)	5.5% (*n* = 45)	n.r.	0.1% (*n* = 1)	0.1% (*n* = 1)	n.r.	10.8% (*n* = 87)	n.r.
*Citrobacter-Enterobacter-Serratia* group (*n* = 94)	n.r.	n.r.	31.9% (*n* = 30)	31.9% (*n* = 30)	22.3% (*n* = 21)	25.5% (*n* = 24)	25.5% (*n* = 24)	5.3% (*n* = 5)	n.r.	3.2% (*n* = 3)	1.1% (*n* = 1)	n.r.	10.6% (*n* = 10)	n.r.
*Proteus-Providencia-Morganella* group (*n* = 226)	n.r.	n.r.	42.0% (*n* = 95)	42.0% (*n* = 95)	40.3% (*n* = 91)	58.6% (*n* = 122)	58.6% (*n* = 122)	14.6% (*n* = 33)	n.r.	n.r.	0% (*n* = 0)	n.r.	5.3% (*n* = 12)	n.r.
*Pseudomonas* spp. (*n* = 218)	n.r.	n.r.	n.r.	12.8% (*n* = 28)	11.9% (*n* = 26)	55.5% (*n* = 121)	56.4% (*n* = 123)	n.r.	n.r.	26.6% (*n* = 25)	23.4% (*n* = 22)	n.r.	41.6% (*n* = 94)	n.r.

Abbreviations: azithromycin (AZI), cefepime (FEP), cefoxitin (FOX), ceftriaxone (CEF), ciprofloxacin (CIP), ceftazidime (CTZ), fosfomycin (FOS), fusidic acid (FAA), imipenem (IMP), levofloxacin (LEV), meropenem (MER), vancomycin (VAN), n.r.: not relevant (antibiotics affected by intrinsic resistance mechanisms); n.t.: not tested.

**Table 7:** Percentage of resistant isolates from inpatient UTIs against WHO Watch antibiotics.

	AZI	FOX	CEF	CTZ	FEP	CIP	LEV	FOS	FAA	IMP	MER	RIF	TOB	VAN
*Staphylococcus* spp. (*n* = 95)	72.6% (*n* = 67)	35.8% (*n* = 34)	n.r.	n.r.	n.r.	77.9% (*n* = 78)	77.9% (*n* = 78)	n.t.	0% (*n* = 0)	35.8% (*n* = 34)	35.8% (*n* = 34)	0% (*n* = 0)	27.4% (*n* = 26)	0% (*n* = 0)
*Enterococcus* spp. (*n* = 907)	n.r.	n.r.	n.r.	n.r.	n.r.	39.3% (*n* = 356)	39.3% (*n* = 356)	n.r.	n.r.	2.6% (*n* = 24)	n.r.	n.r.	n.r.	4.6% (*n* = 42)
*Escherichia coli* (*n* = 1081)	n.r.	n.r.	14.4% (*n* = 156)	14.4% (*n* = 156)	11.8% (*n* = 128)	30.5% (*n* = 330)	30.5% (*n* = 330)	4.7% (*n* = 51)	n.r.	0.2% (*n* = 3)	0.2% (*n* = 3)	n.r.	26.1% (*n* = 282)	n.r.
*Klebsiella* spp. (*n* = 685)	n.r.	n.r.	43.1% (*n* = 295)	43.1% (*n* = 295)	32.2% (*n* = 221)	46.7% (*n* = 320)	45.7% (*n* = 313)	17.4% (*n* = 119)	n.r.	1.3% (*n* = 9)	1.0% (*n* = 7)	n.r.	27.4% (*n* = 188)	n.r.
*Citrobacter-Enterobacter-Serratia* group (*n* = 114)	n.r.	n.r.	34.2% (*n* = 39)	34.2% (*n* = 39)	25.4% (*n* = 29)	17.5% (*n* = 20)	17.5% (*n* = 20)	11.4% (*n* = 13)	n.r.	1.8% (*n* = 2)	0.9% (*n* = 1)	n.r.	13.2% (*n* = 15)	n.r.
*Proteus-Providencia-Morganella* group (*n* = 462)	n.r.	n.r.	68.4% (*n* = 316)	68.4% (*n* = 316)	68.4% (*n* = 316)	50.8% (*n* = 235)	50.8% (*n* = 235)	9.5% (*n* = 44)	n.r.	n.r.	0% (*n* = 0)	n.r.	14.6% (*n* = 68)	n.r.
*Pseudomonas* spp. (*n* = 563)	n.r.	n.r.	n.r.	19.7% (*n* = 111)	16.9% (*n* = 95)	40.1% (*n* = 226)	43.7% (*n* = 246)	n.r.	n.r.	23.9% (*n* = 135)	21.5% (*n* = 121)	n.r.	27.5% (*n* = 155)	n.r.

Abbreviations: azithromycin (AZI), cefepime (FEP), cefoxitin (FOX), ceftriaxone (CEF), ciprofloxacin (CIP), ceftazidime (CTZ), fosfomycin (FOS), fusidic acid (FAA), imipenem (IMP), levofloxacin (LEV), meropenem (MER), vancomycin (VAN), n.r.: not relevant (antibiotics affected by intrinsic resistance mechanisms); n.t.: not tested.

**Table 8:** Percentage of resistant isolates from outpatient UTIs against WHO Reserve antibiotics and rates of MDR and XDR isolates.

	CFT	COL	QPD	LIN	TIG	MDR	XDR
*Staphylococcus* spp. (*n* = 77)	0% (*n* = 0)	n.r.	0% (*n* = 0)	0% (*n* = 0)	0% (*n* = 0)	19.4% (*n* = 15)	0% (*n* = 0)
*Enterococcus* spp. (*n* = 998)	n.r.	n.r.	n.r.	0% (*n* = 0)	0% (*n* = 0)	1.2% (*n* = 12)	0% (*n* = 0)
*Escherichia coli* (*n* = 2384)	n.r.	0% (*n* = 0)	n.r.	n.r.	n.r.	16.6% (*n* = 396)	0% (*n* = 0)
*Klebsiella* spp. (*n* = 806)	n.r.	0% (*n* = 0)	n.r.	n.r.	n.r.	35.9% (*n* = 289)	0% (*n* = 0)
*Citrobacter-Enterobacter-Serratia* group (*n* = 94)	n.r.	0% (*n* = 0)	n.r.	n.r.	n.r.	31.9% (*n* = 30)	0% (*n* = 0)
*Proteus-Providencia-Morganella* group (*n* = 226)	n.r.	n.r.	n.r.	n.r.	n.r.	49.8% (*n* = 112)	0% (*n* = 0)
*Pseudomonas* spp. (*n* = 218)	n.r.	0% (*n* = 0)	n.r.	n.r.	n.t.	14.2% (*n* = 31)	0% (*n* = 0)

Abbreviations: ceftaroline-fosamil (CFT), colistin (COL), linezolid (LIN), tigecycline (TIG), quinpristin-dalfopristin (QPD); n.r.: not relevant (antibiotics affected by intrinsic resistance mechanisms); n.t.: not tested.

**Table 9:** Percentage of resistant isolates from inpatient UTIs against WHO Reserve antibiotics and rates of MDR and XDR isolates.

	CFT	COL	QPD	LIN	TIG	MDR	XDR
*Staphylococcus* spp. (*n* = 95)	0% (*n* = 0)	n.r.	0% (*n* = 0)	0% (*n* = 0)	0% (*n* = 0)	40.0% (*n* = 38)	0% (*n* = 0)
*Enterococcus* spp. (*n* = 907)	n.r.	n.r.	n.r.	0% (*n* = 0)	0% (*n* = 0)	5.4% (*n* = 49)	0% (*n* = 0)
*Escherichia coli* (*n* = 1081)	n.r.	0% (*n* = 0)	n.r.	n.r.	n.r.	16.7% (*n* = 180)	0% (*n* = 0)
*Klebsiella* spp. (*n* = 685)	n.r.	0% (*n*=0)	n.r.	n.r.	n.r.	53.7% (*n*=367)	0% (*n*=0)
*Citrobacter-Enterobacter-Serratia* group (*n*=114)	n.r.	0% (*n* = 0)	n.r.	n.r.	n.r.	31.6% (*n* = 36)	0% (*n* = 0)
*Proteus-Providencia-Morganella* group (*n* = 462)	n.r.	n.r.	n.r.	n.r.	n.r.	69.7% (*n* = 322)	0% (*n* = 0)
*Pseudomonas* spp. (*n* = 563)	n.r.	0% (*n* = 0)	n.r.	n.r.	n.t.	27.2% (*n* = 153)	0.4% (*n* = 2)

Abbreviations: ceftaroline-fosamil (CFT), colistin (COL), linezolid (LIN), tigecycline (TIG), quinpristin-dalfopristin (QPD); n.r.: not relevant (antibiotics affected by intrinsic resistance mechanisms); n.t.: not tested.

## Data Availability

All data generated during the study are presented in this paper.
